# Fibrin glue mediated direct delivery of radiation sensitizers results in enhanced efficacy of radiation treatment

**DOI:** 10.1007/s12672-024-00953-x

**Published:** 2024-04-04

**Authors:** Jane Nguyen, Akhil Chandekar, Sophia Laurel, Jazleen Dosanjh, Keya Gupta, Justin Le, Henry Hirschberg

**Affiliations:** Beckman Laser Institute, University of California, Irvine, CA 92617 USA

**Keywords:** Radiation sensitizer, Fibrin glue, Radiation therapy, Hydrogels, Direct delivery system

## Abstract

**Purpose:**

Radiation therapy (RT) plays an important role in the treatment of glioblastoma multiforme (GBM). However, inherent intrinsic resistance of tumors to radiation, coupled with the need to consider the tolerance of normal tissues and the potential effects on neurocognitive function, impose constraints on the amount of RT that can be safely delivered. A strategy for augmenting the effectiveness of RT involves the utilization of radiation sensitizers (RS). Directly implanting RS-loaded fibrin glue (FG) into the tumor resection cavity would by-pass the blood brain barrier, potentially enhancing the impact of RT on tumor recurrence. This study investigated the ability of FG to incorporate and release, in non-degraded form, the radiation sensitizers 5-Fluorouracil (5FU) and Motexafin gadolinium (MGd).

**Methods:**

FG layers were created in a 24-well plate by combining thrombin, fibrinogen, and 5FU or MGd. Supernatants from these layers were collected at various intervals and added to F98 glioma spheroid cultures in 96-well plates. Radiation was applied either before or after RS application as single or fractionated dosages. Spheroid growth was monitored for 14 days.

**Results:**

Combined treatment of FG-released 5FU and RT significantly inhibited spheroid growth compared to RS or RT as a single treatment. As a free drug, MGd demonstrated its efficacy in reducing spheroid volume, but had diminished potency as a released RS. Fractionated radiation was more effective than single dose radiation.

**Conclusion:**

Non-degraded RS was released from the FG for up to 72 h. FG-released 5FU greatly increased the efficacy of radiation therapy.

## Introduction

Attaining effective treatment for glioblastoma multiforme (GBM) remains an elusive goal. Typically, the management of gliomas necessitates surgical resection of the tumor. Postoperative adjuvant strategies involving standard radiation and chemotherapy often prove ineffective, resulting in an extremely high incidence of tumor recurrence, 80%–90% within 2 cm of the resection boundary [1–3].

Radiation therapy (RT) forms an important modality in postoperative treatment. However, inherent intrinsic resistance of tumors to radiation, coupled with the need to consider the tolerance of normal tissues and the potential effects on neurocognitive function, impose constraints on the amount of RT that can be safely delivered. A strategy for augmenting the effectiveness of RT involves the utilization of radiation sensitizers (RS) [4–7]. These compounds work by amplifying the radiation's capacity to eliminate tumor cells, all the while maintaining the unaffected radio response of normal tissues. Of the several RS compounds explored, 5-Fluorouracil (5FU) and Motexafin gadolinium (MGd) have emerged as promising radio sensitizers, enhancing the efficacy of RT in treating certain cancers. Previous studies have revealed 5FU's and MGd’s ability to modify cancer cell behavior, rendering them more responsive to the damaging effects of radiation [8–11]. Clinical trials however, involving RS for GBM, have thus far yielded disappointing outcomes [12–15]. Since the RS was given intravenously, one explanation for the poor results obtained would be the limited ability of the RS to pass the blood–brain barrier (BBB).

One method for bypassing the BBB is by implanting, during the initial surgical operation, sustained-release hydrogels loaded with therapeutic drugs or nano-agents, as a surgically targeted direct delivery system (DDS) [16–18]. This method of targeted delivery strategy increases the amount of therapeutic material that can successfully reach the remaining infiltrative tumor cells within the brain parenchyma while reducing potential systemic adverse effects. Additionally, the drug is protected from degradation and clearance until released.

Among the suitable hydrogels available, fibrin hydrogel, often called fibrin glue (FG), stands out for its ability to serve as an efficient drug delivery medium. This is primarily owed to its many characteristics that correspond to the needs of this drug delivery method. Notably, FG is a natural compound, has a long clinical history of use in surgical contexts, having been used for decades as a sealant and to achieve hemostasis, is non-toxic and is also easily molded to coat the walls of the irregularly shaped resection cavity, a distinct advantage. As a result, there has been a concentrated effort to investigate FG loaded with therapeutic substances as a localized and regulated release vehicle [19–21].

The objective of this current in vitro study was to examine the capability of FG to act as a DDS for the radiation sensitizers 5FU and MGd. Its ability to enhance the impact of RT, after its release, was evaluated on three-dimensional F98 glioma spheroids.

## Materials and methods

### Cells

The rat glioma line (F98) was obtained from the American Type Culture Collection (Manassas, VA, USA) and maintained in Advanced Dulbecco’s Modified Eagle Medium with high glucose (Life Technologies Corp., Carlsbad, CA, USA), supplemented with 2% fetal bovine serum, 25 mM HEPES buffer (pH 7.4), penicillin (100 μg/mL^−1^) and streptomycin (100 μg/mL^−1^).

### Radiation sensitizers

The chemotherapeutic drug, 5-Fluorouracil (130.078 g/mol) was obtained from Sigma (St. Louis, MO). Motexafin gadolinium (1,148.42 g/mol) was obtained from Pharmacyclics (Sunnyvale, CA). The detailed experimental protocols for each RS are elucidated in the subsequent discussion section.

### Fibrin glue and drug harvest

FG components were obtained from EMD Millipore Calbiochem (Temecula, CA, USA). FG was composed of a 1:1 ratio of fibrinogen and thrombin, with varying concentrations of 5FU or MGd added to the thrombin. 0.2 mL of RS-loaded thrombin was combined with 0.2 mL of fibrinogen in wells of 24-well microplates. The glue gelled for 30 min at 37 °C. The wells were washed twice by filling with 1.5 mL medium and removing after 10 min to remove any free drug. Following, 1.6 mL of drug-free culture medium was added to the well. FG-released RS is defined as FG-RS.

### Direct measurement of FG drug release

Five FG-RS wells were set up and supernatants, consisting of colorless phosphate-buffered saline (PBS) medium, were collected over varying time intervals ranging from 0.25 to 72 h. Fluorescence emission spectroscopy was performed to determine the relative concentration of 5FU in the supernatant. Measurements were conducted with a Cary Eclipse Fluorimeter, using a 1 cm quartz cuvette. The excitation wavelength was set to λ = 254 nm, which corresponds to the maximum absorption of the compound. Fluorescence emission was recorded between λ = 300 nm and λ = 500 nm. The relative concentrations of the drug in the supernatant samples obtained at different time points were determined by comparing the integrated fluorescence intensity near the emission maximum of λ = 440 nm.

### Spheroid formation

F98 cells were used to form spheroids through a modified centrifugation method as previously described [22]. Briefly, 2.5 × 10^3^ F98 cells in 100 μL of culture medium per well were allotted into the wells of ultra-low attachment surface 96-well round-bottomed plates (Corning In., NY).

Each experimental condition was conducted in 2–3 replicate experiments. Each experiment performed contained 12 columns with 6–8 wells per column, resulting in a total of 144 to 288 wells for each experimental condition. Biological replicates were obtained by repeating the entire experiment on different days and maintaining separate flasks of the F98 cells. Technical replicates were achieved by performing multiple wells within the same experiment.

The plates were centrifuged at 500 g for 10 min. Immediately following centrifugation, the tumor cells formed into a disk shape. The plates were maintained at 37 °C in a 5% CO_2_ incubator for 24 h for formation of the usual 3-dimensional spheroid form. The spheroids formed were uniform and about 0.2 mm in diameter. Spheroid volume was calculated based on their measured diameter. The diameter measurements were halved to convert them into radius measurements, denoted as ‘r’. These radii values were used in the calculation as a perfect sphere using the following equation:

$$\frac{4}{3}\prod {r^3}$$

As an example, a spheroid with a diameter of 800 μm has a volume of 280 cubic mm.

### Radiation treatment with free RS and FG-released RS

**Fig. 1:**
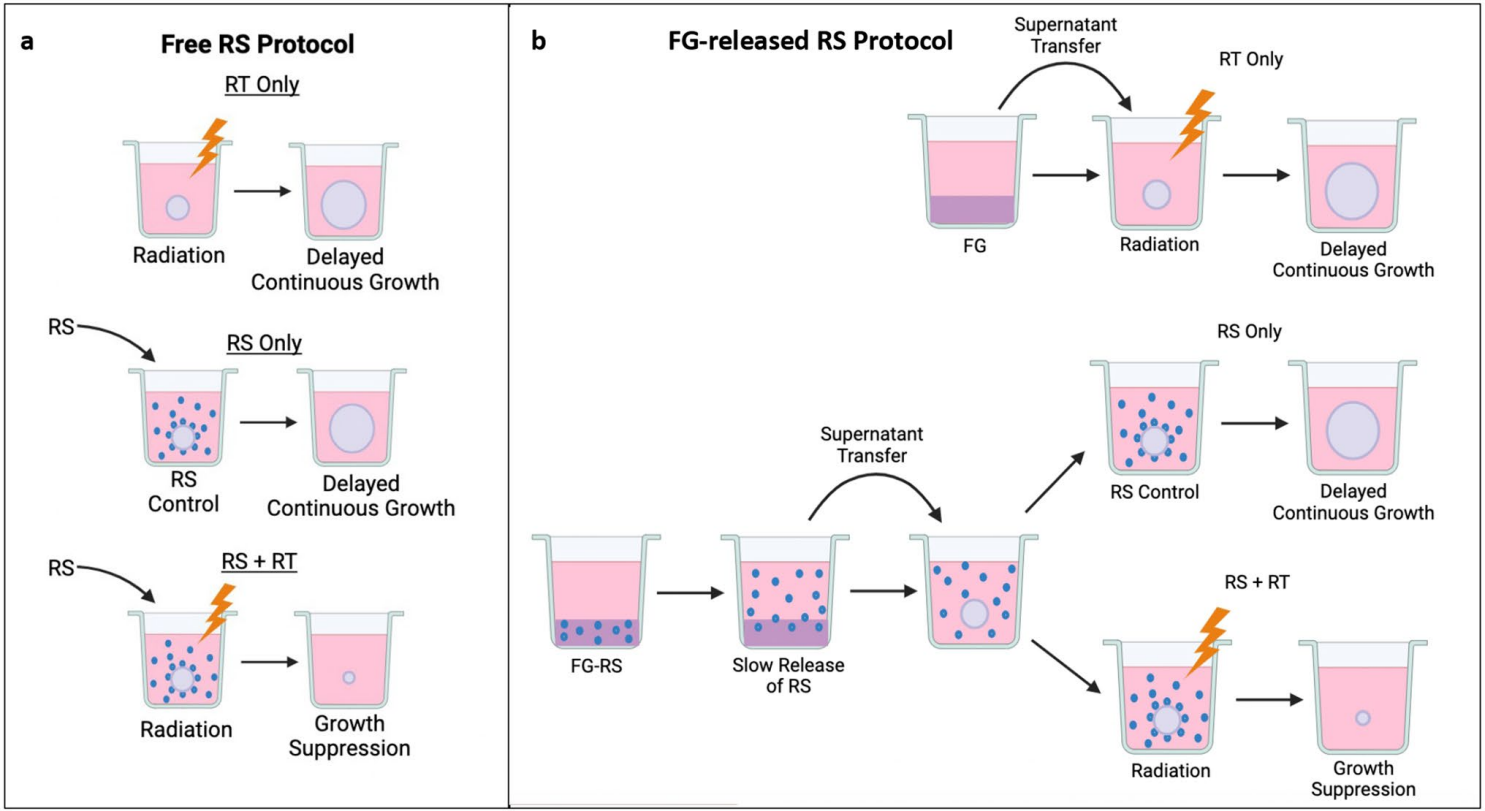
**a** Basic experimental protocol using RS as a free drug. RS (blue dots) added to spheroid cultures. Three arms: (1) RT only, (2) RS only, (3) RS + RT. Spheroids evaluated on day 14. **b** Basic experimental protocol using FG-released RS (FG-RS). Fibrin glue layers, either “empty” or RS-loaded. Covering supernatants harvested, transferred to spheroid cultures. Three arms: (1) RT only, (2) FG-RS only, (3) FG-RS + RT

The basic experimental set up is pictured in Fig. 1a and b. RS as a free drug included three arms: (1) RT only, (2) RS only, (3) RS + RT (Fig. 1a)*.* RS as a FG-released drug included three similar arms: (1) RT only, (2) FG-RS only, (3) FG-RS + RT (Fig. 1b)*.* Free or released RS + RT exposed spheroids received RT either immediately before RS application or 24 h after incubation with the RS. Radiation was administered at a constant dose rate of 1.02 Gy/min with a 50 cm beam distance. Treatment was given at various time intervals to obtain different radiation doses. NTC did not receive either RT or RS. All RT was done in a X-Rad 320 cabinet irradiator (Precision X-Ray Irradiation, Madison, CT, USA). Irradiation voltage and amperage were set at 320 kV and 12.5 mA, respectively. An F2 filter composed of 0.75 mm Tin, 0.25 mm Copper, and 1.5 mm Aluminum was used. After treatment, the spheroids were incubated and monitored for an additional 14 days.

### Statistical analysis

Data analysis and graphing was done using Microsoft Excel. Mean and standard deviation were used throughout. Significance was calculated via Student’s and Welch’s t-test. Two values are considered significantly different when p-values were below 0.05. The following equation determined if the FG-RS + RT effect was synergistic, antagonistic, or additive:

$$\alpha = \frac{{{\text{S}}{{\text{F}}^{\text{a}}} \times {\text{S}}{{\text{F}}^{\text{b}}}}}{{{\text{S}}{{\text{F}}^{{\text{ab}}}}}}$$

SF^a^ and SF^b^ represent the growth following RT and RS as single treatments. SF^ab^, the growth following FG-RS + RT. α > 1, synergistic. α < 1, antagonistic. α = 1, additive.

## Results

### Effects of increasing RT doses on spheroid growth

**Fig. 2:**
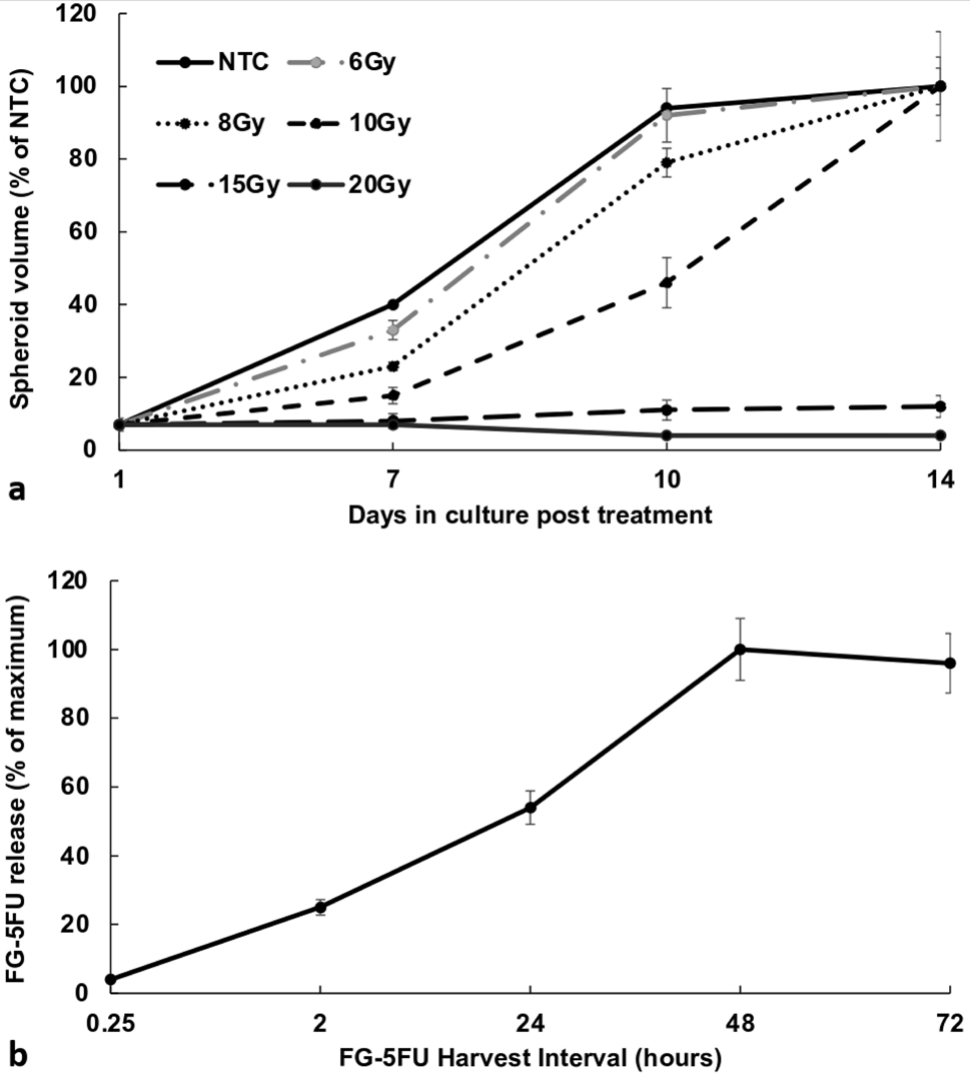
**a** Growth kinetics of F98 spheroids exposed to RT only at doses ranging from 0 to 20 Gy. **b** Direct measurement of FG-5FU released by fluorescence emission spectroscopy. The time course of cumulative release from FG-5FU after 2, 48, 72 h and assayed by drug fluorescence. FG loaded with 1 µg/mL of 5FU. The results are the average of 2 independent experiments and are shown as a % of the maximum release. Error bars represent standard deviation

The growth kinetics of F98 spheroids exposed to increasing doses of RT (0–20 Gy) is shown in Fig. 2a. RT as a single treatment was suboptimal up to 10 Gy, with spheroids usually reaching NTC volume after 14 days in culture. RT alone, however, did produce a growth delay compared to NTC at 8 and 10 Gy. However, radiation doses of 15 and 20 Gy were highly toxic, completely inhibiting spheroid growth even without RS. Assaying these spheroids, after 3 and 4 weeks of incubation, showed no sign of growth (data not shown).

### Determination of FG-5FU drug release kinetics by fluorescence emission spectroscopy

The release profile of 5FU from FG was assayed using drug fluorescence. Here, 1 μg of 5FU was loaded into 0.4 mL of FG. Figure 2b illustrates the results, presenting the release ratio % in the supernatant at harvest intervals of 0.25, 2, 24, 48 and 72 h. The slow, controlled release from FG was demonstrated, with active drugs still being released after 48 h. Based on these results, FG drug harvesting was done at 48 h for all subsequent experiments.

### RT effects on spheroid growth inhibition by 5FU and MGd as free or released drugs acting as RS

**Fig. 3:**
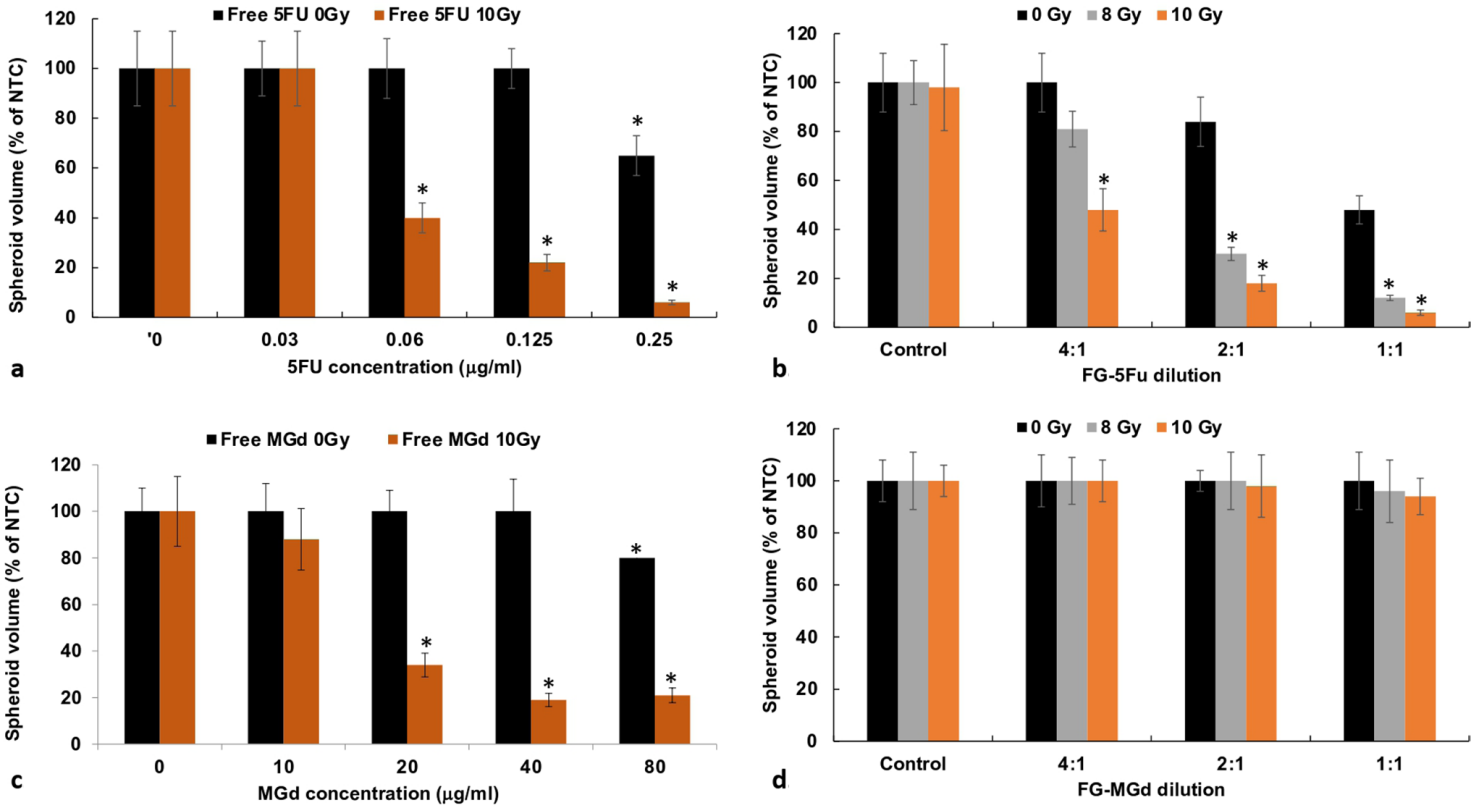
**a** Enhanced RT effects of 5FU as free RS over a range of concentrations, 0–0.25 μg/mL. **b** Effects on spheroid growth of FG-5FU, diluted 1:1, 2:1, 4:1. RT 0, 10 Gy. **c** Enhanced RT effects for MGd over a range of concentrations, 0–80 μg/mL. RT 0 and 10 Gy administered 24 h after RS. **d** FG- MGd diluted 1:1, 2:1, 4:1, and 8:1 with RT 10, 12 Gy Spheroid growth after 14 days. Each data point represents the average volume of 8 spheroids after 2 weeks in culture as a % of non-treated controls. Error bars, standard deviation. * Significant differences (p < 0.05) compared to controls

Figure 3a displays the effects of RT (10 Gy) on spheroid volume for 5FU as a free drug. A significant inhibiting effect was seen with RT in combination with increasing 5FU concentration (0.06–0.25 μg/mL). At a 5FU concentration of 0.25 μg/mL and RT, spheroid volume was 6% of control values after 14 days of growth. In the absence of RT, 5FU showed limited toxicity only at the highest concentration, 0.25 μg/mL.

Loading of FG with 5FU (1 μg) or MGd (40 μg) was done as described in the materials and methods section. Supernatants were harvested after 48 h. As seen in Fig. 3b, FG-5FU combined with 10 Gy RT showed significant growth inhibiting effects compared to RT as a single treatment. At FG-5FU dilutions of 4:1, 2:1, and 1:1 combined with 10 Gy, RT resulted in spheroid volumes of 48%, 18%, and 6% of control volumes, respectively.

Similar results were obtained with MGd (Fig. 3c). MGd as a free drug combined with 10 Gy RT demonstrated significant efficacy at increasing MGd concentrations ranging from 20 to 80 μg/mL, although increasing MGd concentration above 20 μg/mL gave statistically non-significant results (p > 0.05). In contrast, MGd released from FG failed to inhibit spheroid growth combined with 10 Gy RT at all the FG-MGd dilutions examined (Fig. 3d). Based on these results for FG-MGd, all subsequent experiments were done using only 5FU as RS.

### RS + RT kinetics of spheroid growth and light micrograft of spheroids

**Fig. 4:**
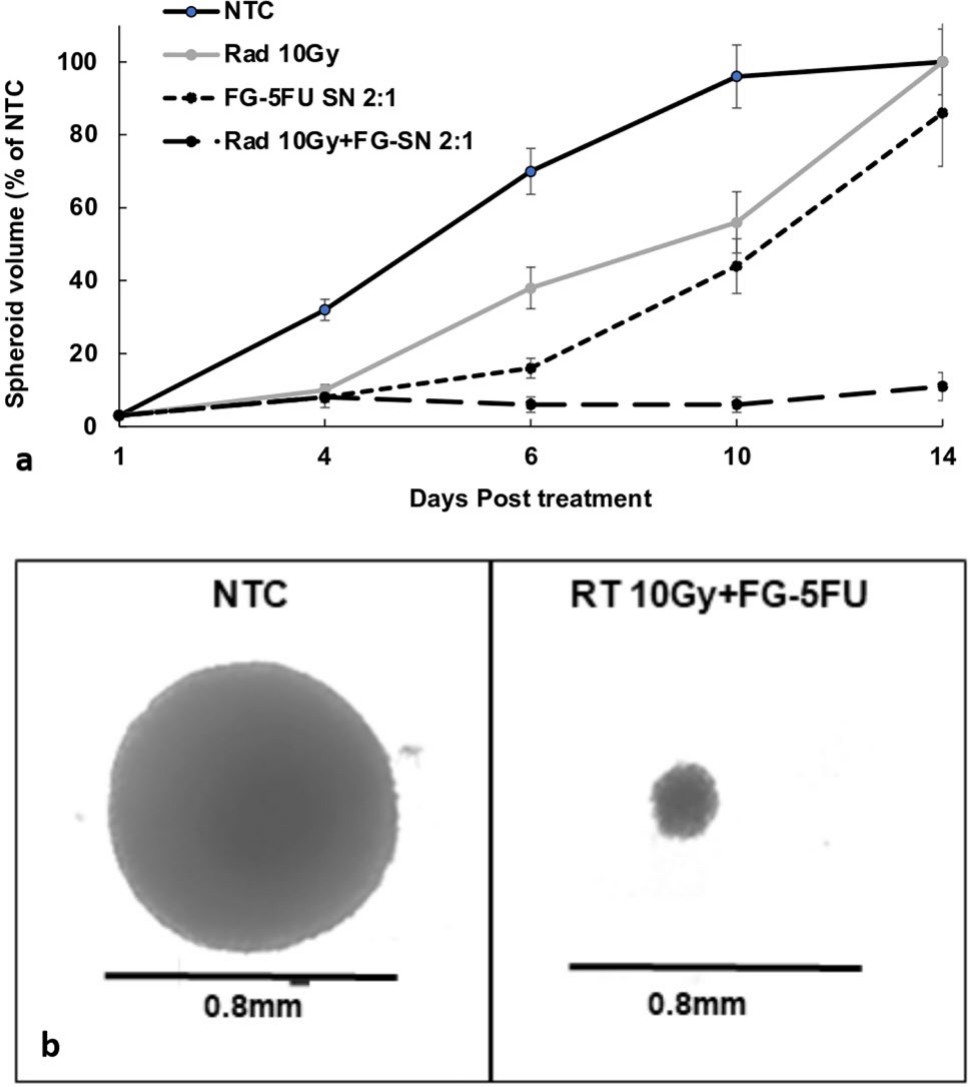
**a** Growth kinetics of spheroid growth following treatment: RT only (10 Gy), FG-5FU only (2:1 dilution), and FG-5FU + RT (2:1 dilution, 10 Gy). Spheroid volume assayed after 4, 6, 10, and 14 days. Each data point represents the average volume of 16 spheroids, from 2 experiments, after 14 days in culture as a % of non-treated controls. Error bars, standard deviation. * Significant differences (p < 0.05) compared to controls. **b** Light micrograph of non-treated control (NTC) and FG-5FU + RT (10 Gy) treated spheroids

The kinetics of a typical spheroid growth pattern, following various forms of treatment, are illustrated in Fig. 4a. RT only (10 Gy) resulted in a significant delay in initial spheroid growth, but they did reach NTC volume after 14 days. When compared to the NTC, FG-5FU treated spheroids reached a volume of 80% of NTC but were still clearly in continuous growth. The most significant finding, however, was that the combined FG-5FU + RT treatment consistently inhibited spheroid growth. Spheroid volumes, at a 2:1 FG-5FU dilution and 10 Gy radiation dose, did not significantly differ from their initial volume on day 1 (p > 0.05). Figure 4b shows a light micrograph of a NTC and a FG-5FU + RT treated (10 Gy) spheroid following 14 days in culture. The FG-5FU + RT treated spheroid is the same size as it was at initiation (0.2 mm) compared to the NTC (0.8 mm). This represents a growth ratio of 64:1.

### RT before and after 5FU addition

**Fig. 5:**
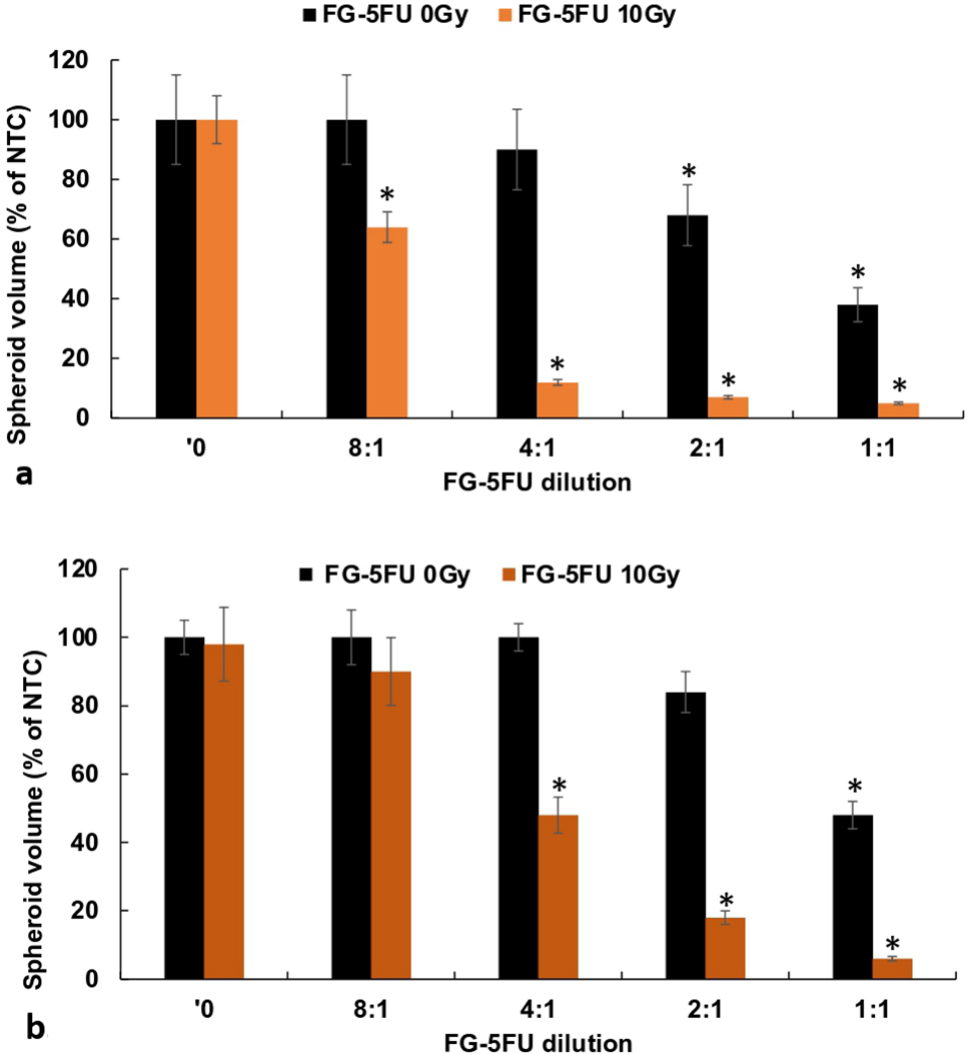
**a** RT before: RT immediately before FG-5FU. **b** RT after: FG-5FU added 24 h before RT. FG-5FU harvested at 48 h, diluted 1:1, 2:1, 4:1, and 8:1. Error bars, standard deviation. * Significant differences (p < 0.05) compared to controls

The effects of RT administration either before or after FG-5FU exposure were evaluated with a protocol where RT was given immediately before FG-5FU addition or where FG-5FU was first added to the spheroids 24 h before RT. The results are shown in Fig. 5, with Fig. 5a representing the RT before and Fig. 5b representing RT after FG-5FU addition. Comparing the two, spheroids subjected to RT prior to FG-5FU exhibited a greater reduction in tumor spheroid size post-treatment. Specifically, at FG-5FU dilution of 2:1, spheroids that were exposed to RT before FG-5FU showed a decrease to 10% of control values while those that underwent RT after FG-5FU addition exhibited a volume of 18% of controls. This was also the case for a 4:1 FG-5FU dilution.

### Single vs fractionated RT

**Fig. 6:**
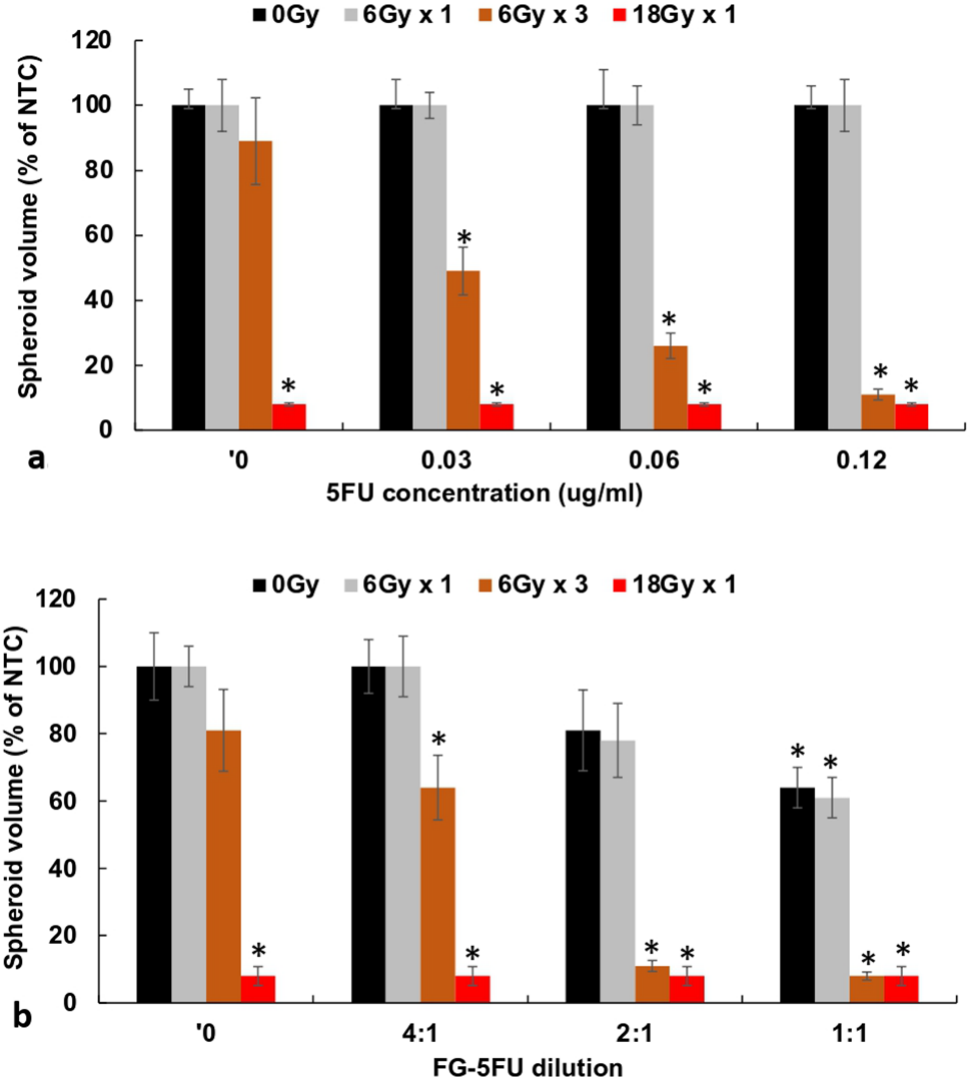
**a** Spheroids incubated with increasing concentrations of 5FU 0, 0.03, 0.06, and 0.12 μg/mL. Radiation 6, 18 Gy administered as single or 3 fractionated doses, 48 h between fractions. **b** FG-5FU diluted 1:1, 2:1, 4:1, and 8:1. Each data point represents the average volume of 16 spheroids, from 2 experiments, after 14 days in culture as a % of non-treated controls. Error bars, standard deviation. * Significant differences (p < 0.05) compared to controls

The fractionation protocol was as follows: free 5FU or FG-5FU added on day 1 and refreshed on day 5. RT (6 Gy) done on day 2, 4, and 6 after spheroid formation. RT in single or fractionated doses for both free 5FU and for FG-5FU is shown in Fig. 6a and b, respectively. Radiation of 6 Gy as a single dose with free 5FU gave no spheroid growth inhibition (Fig. 6a). This was also the case for 6 Gy and FG-5FU which showed non-significant additional growth inhibition (p > 0.05) compared to that obtained with FG-5FU alone. In contrast, fractionated radiation of 6 Gy × 3 induced significant growth inhibition (p < 0.05) for free 5FU over the concentration range of 0.03–0.12 μg/mL and FG-5FU over a range of dilutions 4:1–1:1. A single RT dose of 18 Gy induced complete cessation of spheroid growth irrespective of the concentration of 5FU or the degree of dilution in FG-5FU.

### Synergistic effects of RS + RT

Since RS + RT is a technique that relies on the combination of RS and RT exposure, the resultant toxicity should show more than an additive effect of the single modalities of RS and RT. The degree of synergism was calculated both for free 5FU + RT and for FG-5FU + RT using the formula described in materials and methods. As evidenced from the calculated α values, shown in Table 1, both 5FU + RT and FG-5FU + RT demonstrated a significant synergistic effect (α > 1), particularly at a radiation levels 10 Gy, even at low 5FU and FG-5FU levels.

**Table 1:** Synergistic α values for 5FU free and 5FU FG for increasing RT dose

Radiation	6 Gy	8 Gy	10 Gy
Free 0.06	1.0土0.11	1.1土0.11	2.5土0.18
Free 0.12	1.2土0.14	2.7土0.16	4.4土0.26
FG 4:1	1.0土0.12	1.2土0.10	2.6土0.15
FG 2:1	1.1土0.12	3.0土0.17	6.4土0.29

α value: α > 1, synergistic. α < 1, antagonistic. α = 1, additive

## Discussion

Enhancing anticancer agent delivery across the BBB is important for improving the prognosis of GBM patients. Most published data have been concerned with drug and nanoparticles DDS while RS delivery has been infrequently explored [23–26]. The primary objective of this study was to examine the ability of the hydrogel FG, to act as DDS for the radiation sensitizers 5FU and MGd. The basic premise was that FG was capable of effective uptake and release of RS and that the RS is released in an active form that would increase the efficacy of RT.

The results demonstrated that this was the case for FG-5FU but not for FG-MGd. The observed variance in efficacy between free MGd and FG-MGd might be due to its complex molecular structure. Comparing Fig. 3a with Fig. 3b, there were no significant differences observed in the effects of 5FU, whether administered in its free form or released from the FG on its ability to enhance the effects of RT. The combination of RT with a concentration of 0.125 μg/mL yielded a spheroid volume equal to 22% of control values for free 5FU, in comparison to 18% for RT combined with released FG-5FU at a 2:1 dilution. Using the data in Fig. 3a, the calculated concentration of FG-5FU in Fig. 3b at a dilution of 2:1 is approximately 0.125 μg/mL. Further evidence that FG-5FU is non-degraded can be seen in Table 1. The α values for free (0.12 μg/mL) and FG-5FU (2:1 dilution) are comparable at both 8 and 10 Gy and show a high degree of synergy.

Previous in vitro studies have reported that an optimal response requires 5FU exposure for at least 24 h following RT [8]. The release characteristics of FG as shown in Fig. 1b satisfies this criterion. This is also in agreement with the results presented in Fig. 5a and b. RT immediately before 5FU addition (Fig. 5a) demonstrated a significantly enhanced RT effect compared to 24-h preincubation with 5FU followed by RT (Fig. 5b). As seen in Fig. 4a RT alone, after an initial growth delay, allowed the spheroids to grow to control volumes. This would be equivalent to tumor recurrence in vivo. On the other hand, as seen in Fig. 2a, high radiation doses 15–20 Gy were lethal even in the absence of 5FU. This could be considered an analog to RT toxicity to normal tissue in vivo, the limiting factor for effective treatment.

Since the total release time of RS from FG is measured in days, RT should be administered shortly after surgery. Postoperative external beam RT is typically delayed several weeks to allow wound healing. During this delay, it is highly probable that disease progression occurs so initiating RT shortly after tumor resection would be advantageous. There are several methods of delivering RT shortly after surgery. One such method is the temporary implantation, during surgery, of an after loading balloon catheter implementing fractionated brachytherapy for up to 5 days [27, 28]. A second promising external beam approach is FLASH-RT, characterized by the delivery of ultra-high radiation doses (> 40 Gy/s) in a fraction of a second [29, 30].

## Conclusion

Non-degraded 5FU was released from the FG for up to 72 h. FG-released 5FU greatly increased the efficacy of radiation therapy. Fractionated RT had an increased efficacy compared to an equal radiation dose administered as a single shot. The in vitro results reported here form the basis for translation to in vivo animal experiments and since FG is widely clinically approved, eventually to patient treatment protocols.

## Data Availability

All data supporting the findings of this study are available within the paper.

## References

[1] Chamberlain MC (2011). Radiographic patterns of relapse in glioblastoma. J Neurooncol.

[2] Dobelbower MC, Burnett OL, Nordal RA, Nabors LB, Markert JM, Hyatt MD, Fiveash JB (2011). Patterns of failure for glioblastoma multiforme following concurrent radiation and temozolomide. J Med Imaging Radiat Oncol.

[3] Petrecca K, Guiot MC, Panet-Raymond V, Souhami L (2013). Failure pattern following complete resection plus radiotherapy and temozolomide is at the resection margin in patients with Glioblastoma. J Neurooncol.

[4] Citrin DE, Mitchell JB (2014). Altering the response to radiation: sensitizers and protectors. Semin Oncol.

[5] Allison RR (2014). Radiobiological modifiers in clinical radiation oncology: current reality and future potential. Future Oncol.

[6] Thanekar AM, Sankaranarayanan SA, Rengan AK (2021). Role of nano-sensitizers in radiation therapy of metastatic tumors. Cancer Treat Res Commun.

[7] Gong L, Zhang Y, Liu C, Zhang M, Han S (2021). Application of radiosensitizers in cancer radiotherapy. Int J Nanomedicine.

[8] Byfield JE (1989). 5-Fluorouracil radiation sensitization—a brief review. Invest New Drugs.

[9] Lawrence TS, Davis MA, Tang HY, Maybaum J (1996). Fluorodeoxyuridine-mediated cytotoxicity and radiosensitization require S phase progression. Int J Radiat Biol.

[10] Magda D, Miller RA (2006). Motexafin gadolinium: a novel redox active drug for cancer therapy. Semin Cancer Biol.

[11] Evens AM (2004). Motexafin gadolinium: a redox-active tumor selective agent for the treatment of cancer. Curr Opin Oncol.

[12] Alexander BM, Ligon KL, Wen PY (2013). Enhancing radiation therapy for patients with glioblastoma. Expert Rev Anticancer Ther.

[13] Brachman DG, Pugh SL, Ashby LS, Thomas TA, Dunbar EM, Narayan S, Robins HI, Bovi JA, Rockhill JK, Won M, Curran WP (2015). Phase 1/2 trials of Temozolomide, Motexafin Gadolinium, and 60-Gy fractionated radiation for newly diagnosed supratentorial glioblastoma multiforme: final results of RTOG 0513. Int J Radiat Oncol Biol Phys.

[14] Chang JE, Khuntia D, Robins HI, Mehta MP (2007). Radiotherapy and radiosensitizers in the treatment of glioblastoma multiforme. Clin Adv Hematol Oncol.

[15] Peterson K, Harsh G, Fisher PG, Adler J, Le Q (2001). Daily low-dose carboplatin as a radiation sensitizer for newly diagnosed malignant glioma. J Neurooncol.

[16] Bastiancich C, Danhier P, Préat V, Danhier F (2016). Anticancer drug-loaded hydrogels as drug delivery systems for the local treatment of glioblastoma. J Control Release.

[17] Basso J, Miranda A, Nunes S, Cova T, Sousa J, Vitorino C, Pais A (2018). Hydrogel-based drug delivery nanosystems for the treatment of brain tumors. Gels.

[18] Bastiancich C, Malfanti A, Préat V, Ruman R (2021). Rationally designed drug delivery systems for the local treatment of resected glioblastoma. Adv Drug Deliv Rev.

[19] Anai S, Hide T, Takezaki T, Kuroda J, Shinojima N, Makino K, Nakamura H, Yano S, Kuratsu J (2014). Antitumor effect of fibrin glue containing temozolomide against malignant glioma. Cancer Sci.

[20] Nguyen L, Shin D, Le MT, Potma EO, Idris N, Le JM, Johnson J, Peng Q, Berg K, Hirschberg H (2020). Local drug delivery by fibrin glue for glioma treatment: enhancing drug efficacy by photochemical internalization (PCI). Insights Neurooncol.

[21] Madsen SJ, Devarajan AG, Chandekar A, Nguyen L, Hirschberg H (2023). Fibrin glue as a local drug and photosensitizer delivery system for photochemical internalization: potential for bypassing the blood-brain barrier. Photodiagnosis Photodyn Ther.

[22] Ivascu A, Kubbies M (2006). Rapid generation of single-tumor spheroids for high-throughput cell function and toxicity analysis. J Biomol Screen.

[23] Bastiancich C, Bozzato E, Henley I, Newland B (2021). Does local drug delivery still hold therapeutic promise for brain cancer? A systematic review. J Control Release.

[24] Wang Y, Bastiancich C, Newland B (2023). Injectable local drug delivery systems for glioblastoma: a systematic review and *meta*-analysis of progress to date. Biomater Sci.

[25] Liang HT, Lai XS, Wei MF, Lu SH, Wen WF, Kuo SH, Chen CM, Tseng WI, Lin FH (2018). Intratumoral injection of thermogelling and sustained-release carboplatin-loaded hydrogel simplifies the administration and remains the synergistic effect with radiotherapy for mice gliomas. Biomaterials.

[26] Xie Y, Liu M, Cai C, Ye C, Guo T, Yang K, Xiao H, Tang X, Liu H (2023). Recent progress of hydrogel-based local drug delivery systems for postoperative radiotherapy. Front Oncol.

[27] Johannesen TB, Wayne K, Lote K, Norum J, Henning JR, Tavera K, Hirschberg H (1999). Intracavity fractionated balloon brachytherapy in glioblastoma. Acta Neurochir.

[28] Johannesen TB, Norum J, Lote K, Olson JA, Scheie D, Hirschberg H (2002). A cost-minimizing analysis of standard radiotherapy and two experimental therapies in glioblastoma. Radiother Oncol.

[29] Bourhis J, Montay-Gruel P, Gonçalves Jorge P, Bailat C, Petit B, Ollivier J, Jeanneret-Sozzi W, Ozshain M, Bochud F, Moeckli R, Germond JF, Vozenin MC (2019). Clinical translation of FLASH radiotherapy: why and how?. Radiother Oncol.

[30] Montay-Gruel P, Acharya MM, Gonçalves Jorge P, Petit B, Petridis IG, Fuchs P, Leavitt R, Petersson K, Gondre M, Ollivier J, Moeckli R, Bochud F, Bailat C, Bourhis J, Germond JF, Limoli C, Vozenin MC (2021). Hypofractionated FLASH-RT as an effective treatment against glioblastoma that reduces neurocognitive side effects in mice. Clin Cancer Res.

